# Supplementary material for: To stay or to go: resource diversity alters the dispersal behavior of sympatric cryptic marine nematodes
Source: PeerJ. 2025 Jan 13;13:e18790. doi: 10.7717/peerj.18790 (PMC11737338; doi:10.7717/peerj.18790)
Supplement: Supplemental Information 1 — (A) The numbers of adult nematodes in the local patch at the time of first effective dispersal (mean ± SE) in the local patch for homogeneous patches with the same food treatments in local and distant patches. (B) The numbers of adult nematodes in the local patch at the time of first effective dispersal (mean ± SE) for heterogeneous patches with E. coli in the local patches and different food treatments in the distant patches (n = 4). [file peerj-13-18790-s001.pdf]

Supplementary Material Accompanying the Paper "To Stay or to Go: Resource Diversity Alters the Dispersal Behavior of Sympatric Cryptic Marine Nematodes"

Rodgee Mae Guden [1*], Sofie Derycke [1,2], and Tom Moens [1]

[1]Marine Biology Unit, Department of Biology, Ghent University, Krijgslaan 281/S8, 9000 Ghent, Belgium

[2]Flanders Research Institute for Agriculture, Fisheries and Food (ILVO), Aquatic Environment and Quality, Jacobsenstraat 1, 8400 Oostende, Belgium

[*]rodgeemae.guden@ugent.be

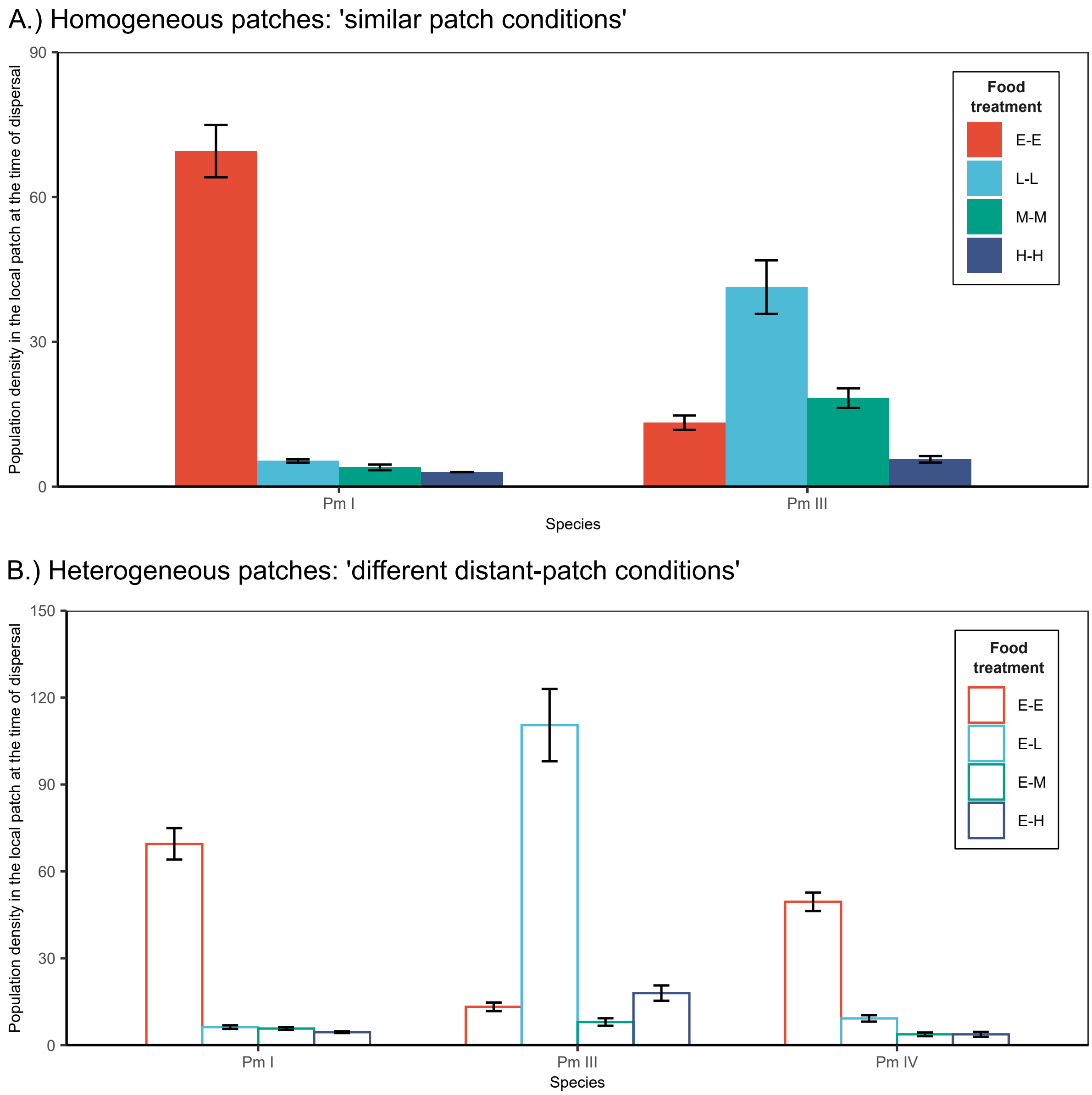

Figure 1: Population density in the local patch at the time of first effective dispersal. (A) The numbers of adult nematodes in the local patch at the time of first effective dispersal (mean ± SE) in the local patch for homogeneous patches with the same food treatments in local and distant patches. (B) The numbers of adult nematodes in the local patch at the time of first effective dispersal (mean ± SE) for heterogeneous patches with *E. coli* in the local patches and different food treatments in the distant patches ($n = 4$).
